# Justice to Homœopaths

**Published:** 1885-12

**Authors:** 


### JUSTICE TO HOMŒOPATHS.

The trustees of the City Hospital of Rochester, N. Y., are considering the advisability of admitting homœopathists to practice in that institution, a resolution proposing to set apart one-half of the hospital for that purpose having been adopted.

